# 2016 Canadian Association of Gastroenterology Educational Needs Assessment Report

**DOI:** 10.1155/2016/6047181

**Published:** 2016-10-27

**Authors:** Kevin Waschke, Karen Sparkes

**Affiliations:** ^1^VP CAG Education Affairs, Canadian Association of Gastroenterology, Oakville, ON, Canada; ^2^National Office, Canadian Association of Gastroenterology, Oakville, ON, Canada

## 1. Introduction

The annual survey of Canadian Association of Gastroenterology (CAG) members' educational needs was conducted via an online survey sent to 1036 CAG members in March 2016. A total of 100 individuals responded to the survey, of which 95 went on to rate educational topics. Similar to previous years, inflammatory bowel disease (IBD) topics were most in demand for future educational events. Other highly rated areas were endoscopic techniques and therapeutics, nutrition in IBD, live endoscopy, celiac disease, upper GI bleeding, NAFLD, nonreflux esophageal disorders, and radiological imaging modalities for GI disorders.

The purpose of the CAG needs assessment was to provide guidance to the Executive and CAG Education Affairs on areas of greatest educational need. Conducting a needs assessment is a requirement for accreditation of educational events in accordance with the Royal College of Physicians and Surgeons of Canada.

## 2. Methods

The members of Education Affairs 2016 include Drs. Robert Berger, Mark Borgaonkar, Herbert Brill, Maria Cino, Samir Grover, Orlee Guttman, Saumya Jayakumar, Charles Menard, Maitreyi Raman, Connie Switzer, Elena Verdu, Catharine Walsh, Kevin Waschke, Geoff Williams, Winnie Wong, Brian Yan, Nauzer Forbes, Premysl Bercik, and Carla Coffin. A subgroup of the committee designed the needs assessment survey, which was a modified version of that used in 2015.

The needs assessment was posted online and members were requested by email to complete the simple “tick box” survey. Data were compiled and analyzed at the CAG National Office.

The survey collected basic demographic information and examined interest in topics for educational events. Respondents were asked to rate their interest in 58 potential topics for educational events using a five-point scale of no interest–minor interest–neutral/not sure–some interest–very interested. The survey also explored use of CAG continuing professional development (CPD) tools.

## 3. Results

A personalized email request sent to CAG members in early March drew 100 respondents of which 95 completed the full survey.

### 3.1. Demographics

Seventy-five percent were male, and, regarding education, 86% held an M.D. or equivalent degree, 16% and 21% held a Ph.D. or M.S. degree, respectively, while 1% held another degree. Of the 99 respondents for whom the question was applicable, the year of medical school graduation was before 1980 for 19%, 1980–1989 for 24%, 1990–1999 for 10%, 2000–2004 for 15%, and 2005 or later for 21% (10% not applicable). For the 99 respondents for whom the question was applicable, the majority were predominantly teaching hospital-based (56%), rather than community-based with (30%) or without (5%) hospital privileges (9% not applicable).

Most replies were from individuals in Ontario (40%), followed by Alberta (18%), British Columbia (16%), and Quebec (10%). Responses were distributed roughly in proportion to provincial population.

Respondents' specialty was identified as adult gastroenterology by 64%, pediatric gastroenterology by 8%, hepatology by 1%, and surgery by 1%. Basic and clinical scientists made up 9% and 3%, respectively, of respondents. Residents and fellows accounted for 7% and “other” roles for 7%.

Regarding where respondents spend their time, 58% identified clinical practice as their primary focus and 14% noted basic research (>50% research). Clinician-teachers (≤50 teaching), clinician-researchers (≤50% research), and clinical research (>50%) formed the next biggest groups at 12%, 10%, and 4% each, respectively. Lastly, 2% were spending their time with “other” duties.

### 3.2. Educational Topics

**Figure 1:**
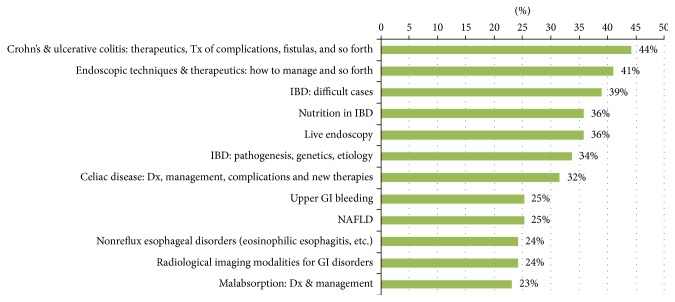
The 12 most popular topics for educational events based on the percent of respondents who were “very interested” in the area (23–44% very interested). Crohn's disease; Dx: diagnosis; NAFLD: nonalcoholic fatty liver disease; GI: gastrointestinal; IBD: inflammatory bowel disease; Tx: treatment.

**Figure 2 fig2:**
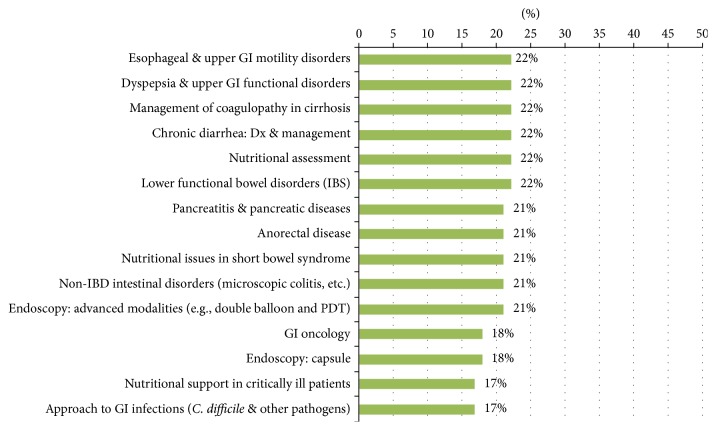
Educational topics in which 17–22% of respondents were “very interested.” GI: gastrointestinal; Dx: diagnosis; IBS: irritable bowel syndrome; IBD: inflammatory bowel disease; GI: gastrointestinal;* C. difficile*:* Clostridium difficile*.

**Figure 3 fig3:**
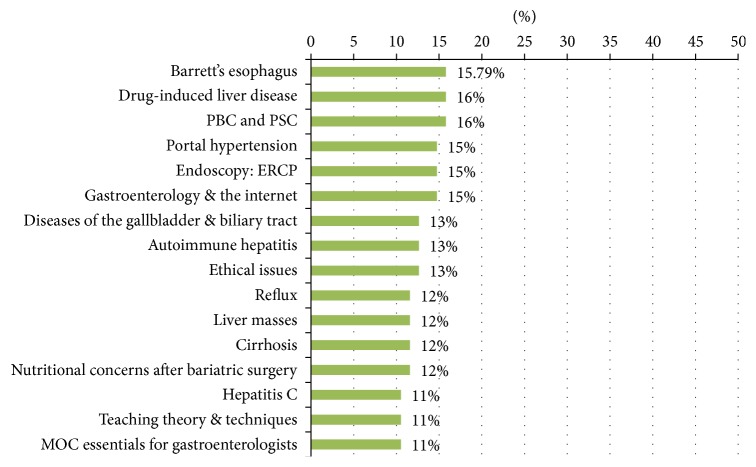
Educational topics in which 11–16% of respondents were “very interested.” PBC: primary biliary cirrhosis; PSC: primary sclerosing cholangitis; ERCP: endoscopic retrograde cholangiopancreatography; MOC: maintenance of certification.

**Figure 4:**
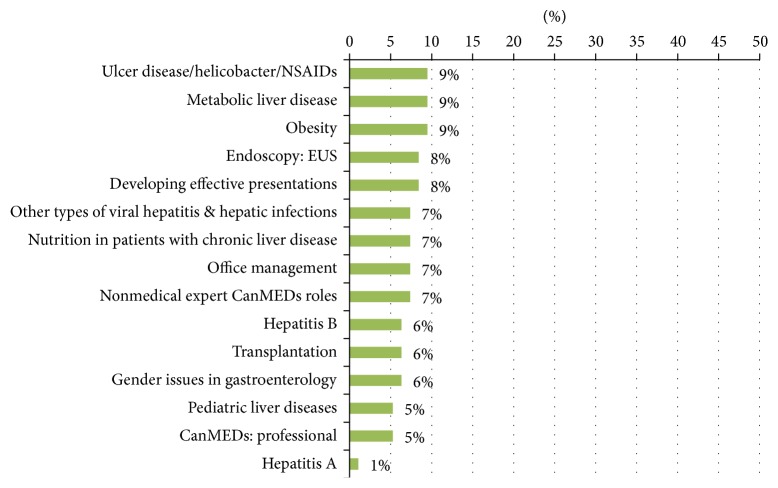
Educational topics in which 1–9% of respondents were “very interested.” NSAIDs: nonsteroidal anti-inflammatory drugs; EUS: endoscopic ultrasound.

The percentage of respondents who were “very interested” in each topic is shown in Figures 1 [Fig fig2] [Fig fig3]–4 for the 58 educational topics surveyed. Consistent with past years, IBD topics remain extremely popular; in addition, endoscopic techniques and therapeutics, nutrition in IBD, live endoscopy, celiac disease, upper GI bleeding, NAFLD, nonreflux esophageal disorders, and radiological imaging modalities for GI disorders were among the most desired educational areas (Figure 1). When examined by various demographic splits (adult versus pediatric gastroenterologist, basic scientists, and teaching hospital versus community hospital respondents), the most desired topics ranked somewhat differently (Table 1). For a basic science symposium, the topics most in demand (percent very interested) were microbiome (42%) followed by mechanisms of inflammation in colitis (27%), mechanisms of disease in IBS (25%), and understanding autoimmunity in relation to gut disease (24%).

### 3.3. CPD Tools

With respect to CAG CPD tools used over the last two years, the one noted to significantly increase knowledge was CAG Consensus Conference Documents (30%), followed by CDDW™ (22%), CAG Skills Enhancement in Endoscopy© (17%), and CAG Visiting Professor Lectures (16%). The tools that significantly changed practice were CAG Consensus Conference Documents (20%), CAG Skills Enhancement for Endoscopy© (17%), and CDDW (14%).

## 4. Discussion

Nine percent of the solicited membership participated in the survey. An ongoing priority for Education Affairs is to develop innovative and easy assessment tools to encourage greater participation by members in order to accurately reflect their educational needs.

IBD remains the highest priority of respondents despite yearly Canadian Digestive Diseases Week (CDDW) sessions in this area since 2002. Apart from IBD, endoscopic techniques and therapeutics, nutrition in IBD, live endoscopy, celiac disease, upper GI bleeding, NAFLD, nonreflux esophageal disorders, and radiological imaging modalities for GI disorders were popular. CAG Consensus Conference Documents have become the CPD tool voted to most significantly change practice (followed by CAG Skills Enhancement for Endoscopy© program). CAG Education Affairs is actively working to increase the quantity and quality of educational materials and programs that members can utilize as part of their ongoing maintenance of certification activities. These findings, along with evaluations of CDDW 2016 and identification of unrecognized educational needs, will form the basis of the 2017 CDDW program.

*Kevin Waschke* *Karen Sparkes*

## Figures and Tables

**Table 1:** Most popular educational topics by respondent subgroup. Crohn's: Crohn's disease; UC: ulcerative colitis; GI: gastrointestinal; IBD: inflammatory bowel disease; IBS: irritable bowel syndrome; NAFLD: nonalcoholic fatty liver disease.

	1st choice (% very interested)	2nd choice (% very interested)	3rd choice (% very interested)	4th choice (% very interested)	5th choice (% very interested)
Gastroenterologists (i) Adult (*n* = 64)	Endoscopic techniques/therapeutics (51.7%)	Crohn's & ulcerative colitis therapeutics; live endoscopy (46.7%)	IBD: difficult cases (45%)	Celiac disease (40%)	Upper GI bleeding (33.3%)

Gastroenterologists (i) Pediatric (*n* = 8)	Nutrition in IBD; Crohn's & ulcerative colitis therapeutics (62.5%)	IBD: pathogenesis, genetics, etiology; IBD: difficult cases (50%)	Pediatric liver diseases; nutritional issues in short bowel syndrome; approach to GI infections; nonreflux esophageal disorders (37.5%)		

Teaching hospital-based (*n* = 55)	Crohn's & ulcerative colitis therapeutics (43.6%)	Nutrition in IBD; endoscopic techniques/therapeutics (41.8%)	IBD: difficult cases; live endoscopy (38.2%)	Celiac disease (30.9%)	Nutritional Issues in short bowel syndrome; nutritional assessment; IBD: pathogenesis, genetics, etiology (29.1%)

Community hospital-based (*n* = 27)	Crohn's & ulcerative colitis therapeutics; NAFLD; endoscopic techniques/therapeutics (48.1%)	IBD: difficult cases (44.4%)	Live endoscopy (40.7%)	Nonreflux esophageal disorders; celiac disease; management of coagulopathy in cirrhosis (37%)	IBD: pathogenesis, genetics, etiology; upper GI bleeding (33.3%)

Basic scientists (*n* = 9)	IBD: pathogenesis, genetics, etiology (44.4%)	GI oncology (33.3%)	Nutrition in IBD (22.2%)	Non-IBD intestinal disorders; obesity; Crohn's & UC; IBD: difficult cases; developing effective presentations (11.1%)	

